# Structural and Spectroscopic Characterization of A Nanosized Sulfated TiO_2_ Filler and of Nanocomposite Nafion Membranes

**DOI:** 10.3390/polym8030068

**Published:** 2016-03-01

**Authors:** Valentina Allodi, Sergio Brutti, Marco Giarola, Mirko Sgambetterra, Maria Assunta Navarra, Stefania Panero, Gino Mariotto

**Affiliations:** 1 Department of Computer Science, University of Verona, Strada le Grazie 15, 37134 Verona, Italy; valentina.allodi@univr.it (V.A.); marco.giarola@univr.it (M.G.); 2 Department of Sciences, University of Basilicata, V.le dell’Ateneo Lucano 10, 85100 Potenza, Italy; sergio.brutti@unibas.it; 3 Department of Chemistry, Sapienza University of Rome. P.le Aldo Moro 5, 00185 Rome, Italy; mirko.sgambetterra@uniroma1.it (M.S.); mariassunta.navarra@uniroma1.it (M.A.N.); stefania.panero@uniroma1.it (S.P.)

**Keywords:** fuel cells, nafion nano-composite membranes, sulfated titanium dioxide, structural (XRD), thermal (TGA) and morphological (TEM) characterization, vibrational spectroscopy, FT-IR absorption and ATR spectroscopy, micro-Raman spectroscopy

## Abstract

A large number of nano-sized oxides have been studied in the literature as fillers for polymeric membranes, such as Nafion^®^. Superacidic sulfated oxides have been proposed and characterized. Once incorporated into polymer matrices, their beneficial effect on peculiar membrane properties has been demonstrated. The alteration of physical-chemical properties of composite membranes has roots in the intermolecular interaction between the inorganic filler surface groups and the polymer chains. In the attempt to tackle this fundamental issue, here we discuss, by a multi-technique approach, the properties of a nanosized sulfated titania material as a candidate filler for Nafion membranes. The results of a systematic study carried out by synchrotron X-ray diffraction, transmission electron microscopy, thermogravimetry, Raman and infrared spectroscopies are presented and discussed to get novel insights about the structural features, molecular properties, and morphological characteristics of sulphated TiO_2_ nanopowders and composite Nafion membranes containing different amount of sulfated TiO_2_ nanoparticles (2%, 5%, 7% *w*/*w*).

## 1. Introduction

Among the different polymer electrolytes proposed as proton exchange membranes (PEM) for fuel cell (FC) applications, one of the best choices is represented by perfluorinated polymers, such as Dupont’s Nafion^®^, due to their high proton conductivity and the excellent mechanical and chemical stability. Unfortunately, ionic conduction of Nafion is strongly dependent on the membrane hydration, and a sharp decrease in conductivity is observed at working temperatures above 80–100 °C due to dehydration and polymer structure relaxation. An upgrade of the Nafion properties in critical conditions (high temperature, low relative humidity) can be achieved by loading the pristine Nafion with hydrophilic inorganic acids, which can both act as a water reservoir, into the polymer matrix (thus increasing the membrane water uptake) and, in virtue of their acidity, provide additional pathways for proton hopping inside the polymer. Among the inorganic acids, sulfated transition-metal oxides have become the subject of intensive studies, due to the high stability and extraordinary acidity of some of these compounds, as, for instance, sulfated zirconium oxide [1,2,3], sulfated tin oxide [4,5], and sulfated titanium oxide [6]. The latter has been widely studied in the past as a catalyst and as a proton conductor [7,8]. Recently, sulfated titania nanoparticles have been added to various polymers to form composite membranes with improved thermal and mechanical properties and enhanced proton conductivity [9].

This work reports on the results of a multi-technique characterization of nanosized sulfated TiO_2_ powders obtained through a direct one-step synthesis and their incorporation as fillers in Nafion-based polymer electrolytes. To the best of our knowledge, the inclusion of one-step synthesized S-TiO_2_ in Nafion membranes has never been reported before, with the exception of our recent work where water dynamics inside S-TiO_2_-added Nafion membranes were investigated by NMR spectroscopy [10]. In the present paper, structural features, molecular properties, as well as morphological characteristics of TiO_2_ nanopowders, both before and after the incorporation in Nafion membranes, have been investigated by a variety of advanced techniques, such as synchrotron X-ray diffraction (XRD), transmission electron microscopy (TEM), thermogravimetry (TGA), and vibrational spectroscopies (both Raman scattering and infrared absorption), paying particular attention to the effects caused by their exposure to a high humidity. Finally, the homogeneity degree of both structure and morphology of composite Nafion membranes with different amounts of sulfated TiO_2_ nanoparticles (2%, 5%, 7% *w*/*w*) has been probed in the micrometric scale by Raman mapping. Our goal is to illustrate how the incorporation of sulfated nanostructured titania into Nafion membranes alters the local environment of the ionic and hydrophobic domains of the membranes in terms of bonding and molecular interactions.

## 2. Materials and Methods

Sulfated titania (S-TiO_2_) was obtained through a one-step sol-gel procedure by adapting the synthesis proposed by Swaminathan and co-workers [11]. A solution of titanium isopropoxide in 2-propanol was used as Ti source and an aqueous sulfuric acid solution was adopted for both hydrolysis and sulfating processes. H_2_SO_4_ (0.5 M; 6.4 mL) was added to a mixture of 12.5 mL of titanium(IV) isopropoxide (Sigma-Aldrich, St. Louis, MO, USA) and 100 mL of 2-propanol (Sigma-Aldrich, St. Louis, MO, USA) with vigorous stirring. After 2 h, the solution was filtered, then calcined for 3 h at 400 °C to obtain an inorganic powder. Plain and composite Nafion membranes were prepared according to a solvent-casting procedure already reported in a previous work [3]. A proper dispersion of commercial Nafion (5 wt % in water/alcohol, E.W. 1100, Ion Power, GmbH) was treated with *N*,*N*-dimethylacetamide at 80 °C in order to replace the solvents. As for the composite membranes, the inorganic powder was added to the final Nafion solution and stirred to homogenize the dispersion. Filler concentrations of 2%, 5%, and 7% *w*/*w* of S-TiO_2_ with respect to Nafion content were chosen. Each mixture was casted on a Petri dish and dried at 100 °C to obtain self-standing membranes. After that, dry membranes were hot-pressed at 175 °C and 50 atm for 15 min in order to improve their thermo-mechanical properties. They were finally activated by immersion in a boiling solution of hydrogen peroxide (3%), sulfuric acid (0.5 M), and water. All of the membrane samples were stored in distilled water after preparation. Membrane samples are labeled here as N for plain Nafion and as nTiO_2_-S (*n* = 2, 5, 7) for composite Nafion membranes filled by different nominal amounts of sulfated inorganic powder. Table 1 summarizes the samples investigated in this work and the acronyms used to identify them.

Synchrotron X-ray diffraction experiments were carried out at the ELETTRA synchrotron radiation source (MCX beamline) on capillarized S-TiO_2_ powders. An X-ray wavelength of 1.204 Å has been used in a diffractometer equipped with a four-circle Huber goniometer (2θ precision better than 0.0001°) in full circle configuration. The diffraction spectra were recorded in the 15–67 degree 2-theta (2θ) range, with a step size of 0.01 degrees and a time per step of two seconds. The structural refinement has been carried out by the GSAS code [12] starting from the anatase polymorphic lattice of TiO_2_ [13].

Transmission electron microscopy measurements were performed by a FEI G2 20 HR-TEM instrument equipped with a LaB6 electron beam source and two 2D flat cameras (low-resolution and high-resolution). Samples have been suspended in acetone in an ultrasonic bath and dispersed on copper holey carbon film grids for observation.

Thermal properties of the powders were evaluated by means of thermogravimetric analysis (TGA) performed in air flux (60 mL·min^−1^) at a heating rate of 5 °C·min^−1^ with a TGA/SDTA 851 Mettler-Toledo (Greifensee, Switzerland). In order to investigate the hydro-thermal stability of the synthesized oxide, TGA measurements were performed on both pristine and hydrolyzed powders, these latter obtained according to the following protocol. S-TiO_2_ powder was dispersed in boiling water (1 mL of water for 1 mg of powder) under vigorous stirring for 1 h, filtered, and washed three times with cold water, and finally calcined at 400 °C for 3 h.

Vibrational characterization of both sulfated titania nanopowders and composite membranes was carried out by means of FT-IR and Raman spectroscopy measurements. FT-IR spectra were obtained at room temperature, using a JASCO spectrometer (FT/IR-660 plus, JASCO, Easton, MD, USA) equipped with a Tri-Glycine-Sulfate (TGS) detector, either in attenuated total reflection (ATR) configuration (using a germanium crystal for both kinds of samples, spectral range between 4000 and 900 cm^−1^), or in transmission configuration in KBr pellets. All of the IR spectra were recorded with a resolution of 4 cm^−1^, and a polystyrene film was used as reference for wavenumber calibration. In order to allow for a better comparison between different samples, a proper baseline has been carefully subtracted from each FT-IR spectrum. Prior to the measurement each membrane was dried in vacuum for 30 min to avoid the occurrence of spectral differences between the samples due to a different hydration degree.

Micro-Raman spectroscopy measurements were carried out in backscattering geometry at room temperature using, in turn, two different Horiba-Jobin Yvon micro-sampling spectrometers (Horiba, Kyoto, Japan): a LABRAM HR, consisting of a single monochromator, for spectra detection extended to a very high wavenumber region and a triple-axis monochromator (model T64000) in order to probe the spectral region below 200 cm^−1^ down to a few wavenumbers from the laser excitation line. The single monochromator spectrometer was equipped with a He–Ne laser as excitation source (632.8 nm) and a notch filter for the Rayleigh line cut-off. The scattered radiation was dispersed by a removable diffraction grating having 600 or 1800 lines/mm and detected at the spectrograph output by a multichannel device, a CCD with 1024 × 256 pixels, cooled by liquid nitrogen, and with its maximum efficiency occurring in the red region. The spectral resolution was about 1 cm^−1^/pixel when the 1800 lines/mm grating was used, while the spectral limit on the side of low wavenumbers, due to the notch filter, was about 200 cm^−1^. In order to investigate the low-wavenumber spectral region, the triple-axis monochromator, set in double subtractive/single configuration, and equipped with holographic gratings having 1800 lines/mm, was exploited. For most measurements carried out by means of this spectrometer, the excitation source was the 514.5-nm line of a mixed Ar-Kr ion gas laser. The scattered radiation detection was ensured by a multichannel detector an open-electrode CCD, consisting of a matrix of 1024 × 256 pixels, cooled by liquid nitrogen, whose maximum efficiency occurred in the green/yellow region. In these conditions the spectral resolution was about 0.4 cm^−1^/pixel. Both micro-Raman setups were coupled to a camera which allowed for the exploration and selection of the sample region worth to be measured. Independently of the spectrometer used to carry out the Raman measurements, the spectra were obtained by focusing the laser beam onto a spot of about 2 μm in size through a long-working distance 50× objective, with medium numerical aperture (N.A. = 0.5), or through a 80× objective with high numerical aperture (N.A. = 0.9). The laser power at the samples surface was kept below 5 mW. All the spectra were calibrated in wavenumber using the emission lines of an Ar spectral lamp. In order to verify the spectra reproducibility over the sample surface, repeated micro-Raman spectra were carried out under the same experimental conditions from different points of the investigated sample. The recorded spectra were processed to remove artifacts due to cosmic rays, while the luminescence background, consisting of a continuous line, underlying the overall Raman spectrum and having both the shape and the intensity dependent of the probed sample region, was subtracted before starting the analysis of the experimental data.

## 3. Results and Discussion

### 3.1. Structural and Thermal Characterization of the Sulfated Powders

**Figure 1:**
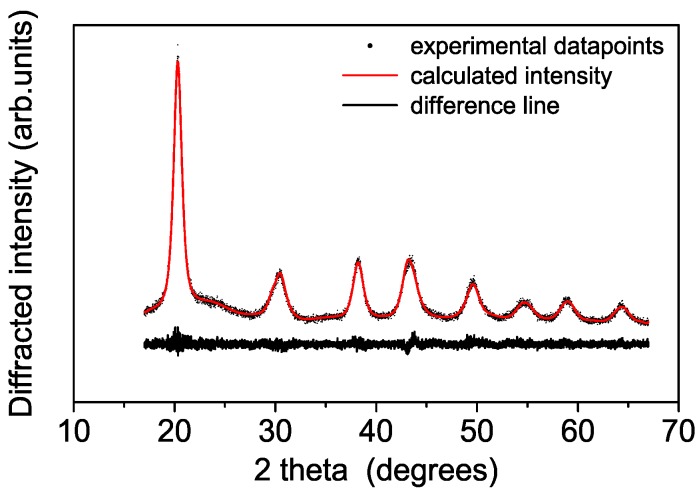
Synchrotron diffraction patters of the synthesized samples fitted by Rietveld method using the GSAS software.

A typical diffraction pattern of the synthesized powders recorded at the synchrotron radiation source is shown in the Figure 1. The synthesized material consists of nanosized nanoparticles of anatase as confirmed by synchrotron diffraction. The Rietveld refinement suggests a slightly deformed trigonal anatase lattice with crystal parameters of *a* = 3.791 Å and *c* = 9.439 Å to be compared to *a* = 3.784 Å and *c* = 9.514 Å literature values, respectively (convergence parameters Rwp = 4.9% RF_2_ = 1.0%, DWd = 1.74) [13,14]. Occupancies and Debye-Waller factors have been fixed in the refinement (x(Ti) = x(O) = 1; B(O) = 2.0; and B(Ti) = 1.0). The refined crystal structure is in a satisfactory agreement with literature data, e.g., the atomic site position of oxygen (x = 0.926) to be compared with that of [13,15,16]. The undulations of the background suggest the presence also of minor content of nanosized particles with TiO_2_-B structure [17,18,19]. The final crystallite size obtained by size-strain analysis suggests a diameter of about 10.1 ± 0.6 nm.

**Figure 2:**
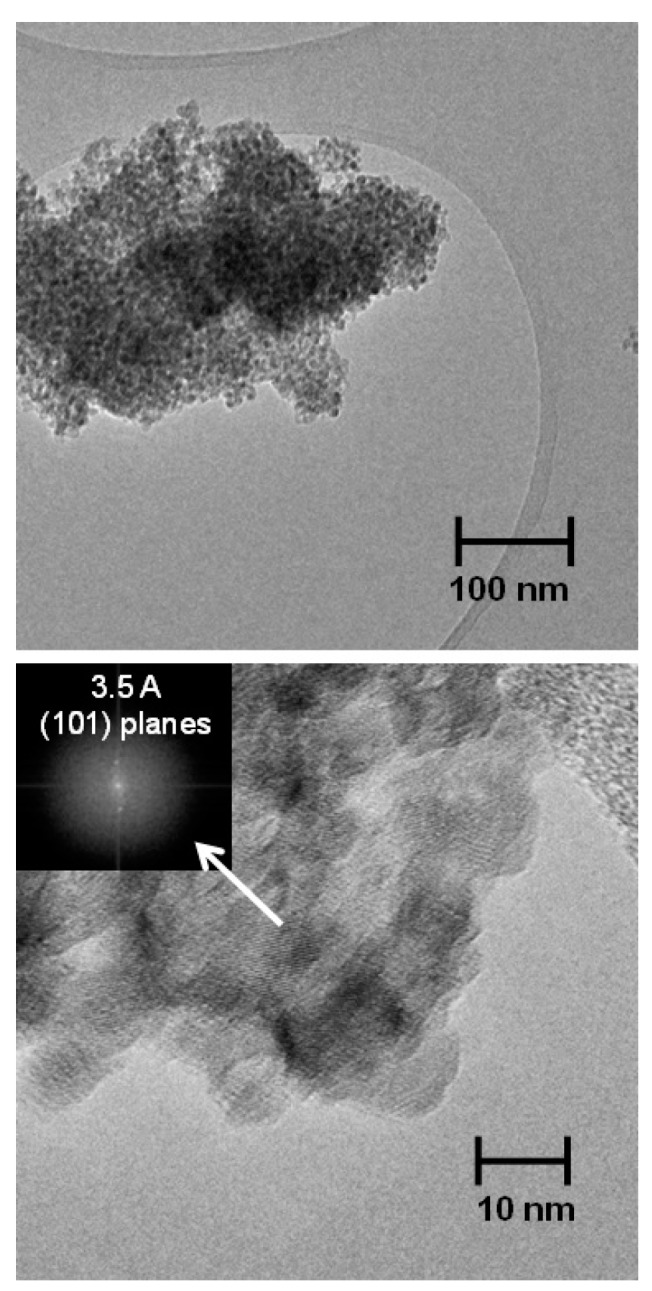
Transmission electron micrographs of the synthesized powders. In the inset of the bottom micrograph a FFT pattern of the periodic arrangement of the crystal planes is shown with an indexing for the trigonal anatase lattice.

The morphology of the S-TiO_2_ ceramic material has been investigated by TEM: two typical micrographs at different magnifications are presented in Figure 2. The low magnification TEM image highlights a highly homogeneous sample constituted by nanometric round-shaped particles of very similar size. The powder is morphologically very pure: apparently no contaminations by larger particles, chunks, or other morphologies can be observed. The size distribution obtained by analyzing 10 micrographs and more than 500 particles by using the ImageJ software [20,21] indicates a mean diameter of about 7.6 ± 2.5 nm in fair good agreement with the value derived from XRD size-strain analysis (10.1 ± 0.6 nm). High-resolution TEM imaging confirms the uniform morphology of the nanoparticles and evidences their crystalline nature, too. Diffraction fringes have been observed throughout the entire sample and the corresponding Fast Fourier Transform (FTT) pattern easily indexed to the crystal lattice planes of anatase TiO_2_ [13].

**Figure 3:**
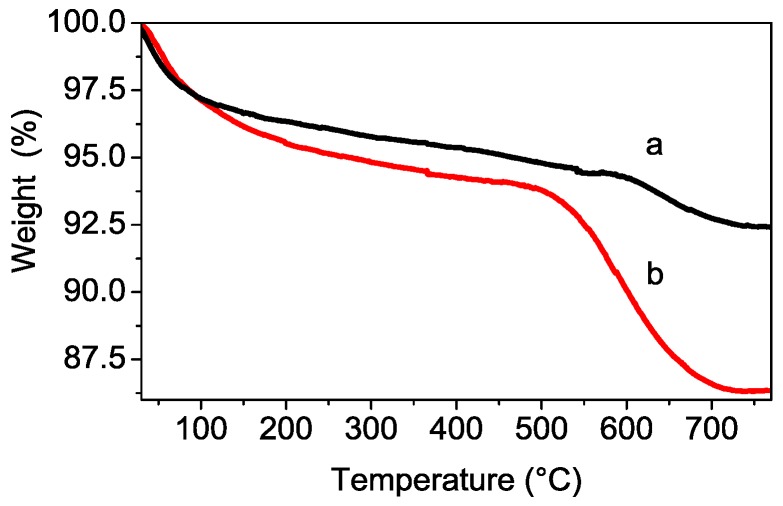
TGA of hydrolyzed (**a**) and pristine (**b**) powders.

The thermal response of S-TiO_2_ is reported in Figure 3: in addition to the pristine S-TiO_2_ material, a second sample has also been studied by TG, namely hydrolyzed-S-TiO_2_. The latter sample has been obtained by a drastic hydrolysis treatment after the standard synthesis, as reported in the experimental section, in order to highlight eventual losses of weakly bonded and physisorbed sulfate surface groups. Both pristine (red curve) and hydrolyzed (black curve) samples present two main weight losses. The first loss, starting just above room temperature until about 500 °C, can be ascribed to removal of water and surficial OH^−^ anions, while the second main loss, occurring above 500 °C, is due to thermal decomposition of SO_4_^2−^ groups. It is worth noticing that a high sulfation degree is detected in pristine S-TiO_2_ powder (*i.e.*, *ca.* 8%) confirming the effectiveness of the synthetic route. Moreover, a certain extent of sulfation is retained after the severe hydrothermal stability test. Indeed, a weight loss of about 2% is observed for the hydrolyzed sample above 500 °C.

**Figure 4:**
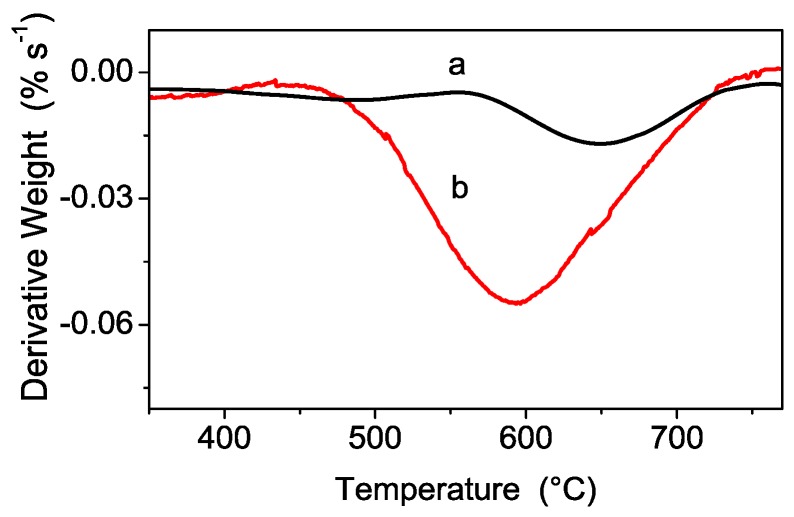
DTG curves of hydrolyzed (**a**) and pristine (**b**) powders.

Derivative thermal gravimetric (DTG) curves of pristine and hydrolyzed powders are reported in Figure 4 in the 300–750 °C temperature region in order to better highlight the different thermal processes of sulfate decomposition occurring in the two samples. A shift of the derivative peak minimum towards higher temperature occurs when moving from pristine (*ca.* 600 °C, red curve) to hydrolyzed (*ca.* 650 °C, black curve) powder. This shift can be explained by assuming the presence of differently-bonded sulfate species: the weakly bonded ones, decomposing at lower temperature, are hydrolyzed, whereas the more strongly-bonded sulfate species are retained even at higher temperature as evidenced by their DTG response.

### 3.2. Vibrational Characterization of the Synthesized Powders

**Figure 5:**
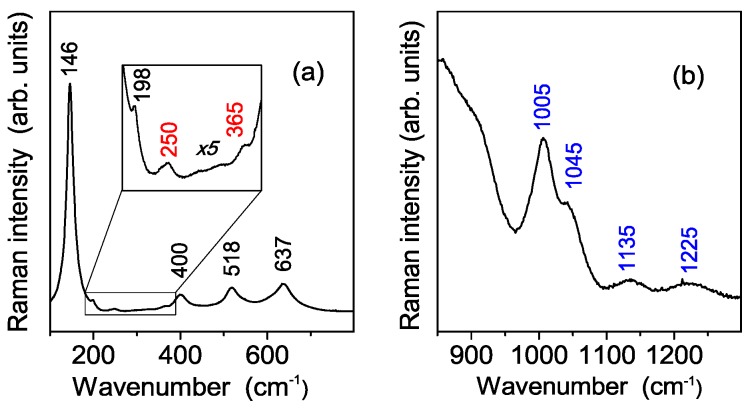
Experimental Raman spectra of the synthesized sample showing typical features of TiO_2_ (panel **a**) and of sulfate group SO_4_^2−^ (panel **b**). The details about the spectra detection are provided in the text.

The vibrational characterization of the synthesized sample was carried out both by Raman and Infrared absorption spectroscopy. A typical Raman spectrum of the S-TiO_2_ powder carried out in the low wavenumber region is shown in the Figure 5a. This spectrum consists of several Raman bands, having quite different spectral amplitude. At first sight it looks very similar to that of nanocrystalline anatase TiO_2_ reported in literature [22], thus indicating that this titania phase is the paramount component of the synthesized powders. However, a deeper insight also reveals the occurrence of some weak Raman modes besides the five ones of anatase TiO_2_, which suggest the presence of a second minor component, probably related to a TiO_2_-B phase, as revealed by the x-ray diffraction results. Therefore, in order to discuss in depth the Raman spectrum measured from the synthesized nanopowders, we shall refer to both of these crystalline titania phases.

Crystalline anatase TiO_2_ has a tetragonal structure which belongs to the space group D_4h_^19^ (I41/amd). Among them six modes are Raman active (1 A_1g_, 2 B_1g_ and 3 E_g_). In micro-crystalline TiO_2_ anatase they occur at about 143 cm^−1^ (E_g_), 198 cm^−1^ (E_g_), 395 cm^−1^ (B_2g_), 512 cm^−1^ (A_1g_), 518 cm^−1^ (B_1g_), 639 cm^−1^ (E_g_) [23]. All of these vibrational modes are present in the spectrum of S-TiO_2_ powders (see Figure 5a), although slightly shifted in wavenumber with respect to single crystal due to the nanocrystalline character of our powders. On the other hand, TiO_2_-B phase is characterized by four formula units per unit cell and, thus, a total of 36 vibrations, among which 12 A_g_ and 6 B_g_ are Raman active modes [24,25]. However, only two TiO_2_-B modes are unambigously observed in S-TiO_2_ Raman spectra, respectively at about 250 and 365 cm^−1^ (see Figure 5a, quoted in red). The missing peaks of this titania phase are probably hidden under the much stronger ones due to anatase. As for the Raman spectra of the S-TiO_2_ powder recorded in the higher wavenumber region, Figure 5b shows the spectral features related to sulfate functionalization: the peak at about 1005 cm^−1^, is assigned to the stretching mode ν_1_ of the SO_4_^2−^ groups, while the three bands (one of which occurring at about 1045 cm^−1^ and two weaker at about 1135 and 1225 cm^−1^) are associated to the splitting of the ν^3^ mode of the SO_4_^2−^ units. The splitting, due to a lowering of the free SO_4_^2−^ anion symmetry, suggests the formation of bidentate sulfate groups coordinated to TiO_2_ nano-particles [26]. Raman spectroscopy therefore confirms that the sample consists of a predominant phase, *i.e.* anatase, and a minor component, *i.e.*, TiO_2_-B, in fair agreement with X-ray diffraction results. Moreover, it clearly reveals the sulphated functionalization of the synthesized powders.

**Figure 6:**
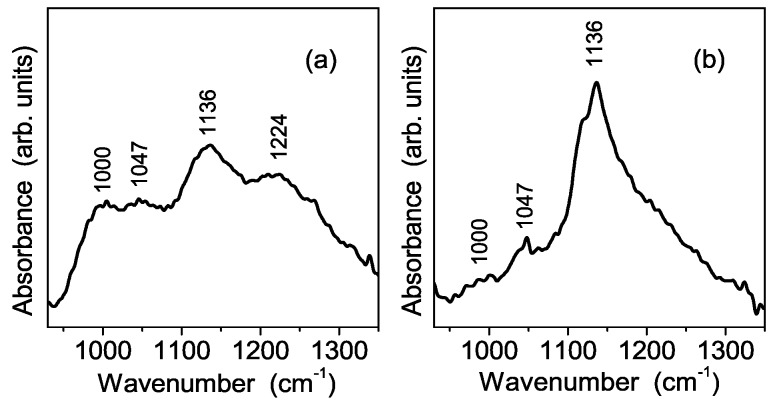
FT-IR ATR spectra of the synthesized sample showing typical features of the sulfate group SO_4_^2−^ recorded before (panel **a**) and after (panel **b**) the storage in high relative humidity environment. The details about the spectra detection are provided in the text.

The vibrational spectrum of sulfate groups of the S-TiO_2_ powder was also detected by ATR FT-IR spectroscopy. Figure 6a clearly shows the occurrence of the ν_1_ vibrational mode at about 1000 cm^−1^ and the three ν^3^ modes at about 1047, 1136 and 1224 cm^−1^, respectively. The number and position of these modes fairly correlate with the above Raman spectroscopy findings, as well as with the observations of Arata and Hino [27] who attributed them to bidentate sulfate coordination at the titania surface.

**Figure 7:**
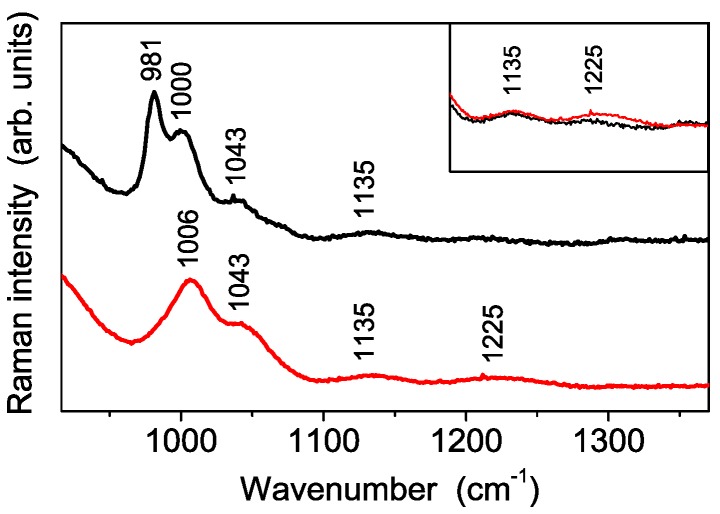
Raman spectra of the synthesized sample before (red) and after (black) the storage in high humidity environment. Inset: direct comparison of the 1225 cm^−1^ peak intensity.

In order to investigate the nanopowder interaction with water, and, at the same time, to simulate the condition of a Nafion membrane in a working fuel cell, samples were stored in a high relative humidity (RH) environment (close to 100% RH) for at least 12 h. The FT-IR spectra recorded on the powder after the exposure to moisture, shown in Figure 6b, shows evidence of remarkable changes of the spectral features with regard to both their number and their relative intensity. In particular, the disappearance of the mode at about 1224 cm^−1^ indicates a different arrangement of SO_4_^2−^ groups in presence of a higher water content, which turns out to promote the switch from a bidentate coordination to a monodentate one of the same groups inside S-TiO_2_ nanoparticles. This change of the sulfate coordination suggests the occurrence of an interaction mechanism between S-TiO_2_ and water molecules similar to that proposed by Bolis *et al.* for sulfated ZnO_2_ [27]. Similar changes on vibrational spectrum of S-TiO_2_ powders exposed to high relative humidity are observed by Raman spectroscopy, which in addition reveals the occurrence of an extra peak at 981 cm^−1^ (Figure 7). This is attributed to the stretching vibrational mode of a quasi-isolated SO_4_^2−^ ion [28,29], which is not detected by IR spectroscopy for symmetry reasons. The Raman spectrum evolution is in accordance with TGA results obtained on the sample before and after the hydrolysis. Therefore, the picture emerging from both Raman and TGA measurements is the following: due the hydrolysis treatment a part of the sulfate groups is released, and, in a high RH environment, this part of sulfate groups shows the spectrum typical of isolated SO_4_^2−^. Moreover, the sulfate ions having a monodentate coordination in the high RH conditions most probably represent the remaining fraction after the hydrolysis treatment.

### 3.3. Vibrational Characterization of Composite Membranes

**Figure 8:**
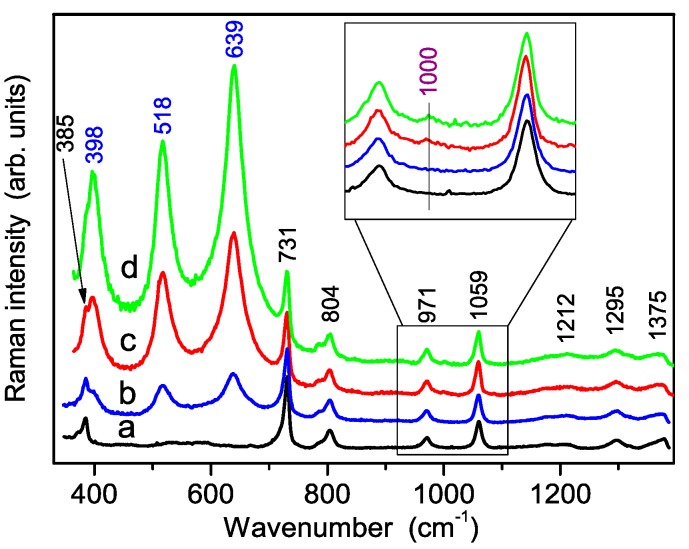
Experimental Raman spectra, carried out in the high wavenumber region, of pure Nafion membrane (**a**) and of three composite membranes with increasing amounts of TiO_2_ nanopowders: 2% (**b**) 5% (**c**) and 7% (**d**). The inset report the spectra of SO_4_^2−^ groups in the region 950–1090 cm^−1^ after proper magnification.

The vibrational properties of Nafion membranes (pure and composite with three different amounts of filler) were also investigated. The related Raman spectra, observed in the wavenumber region above 380 cm^−1^, are reported in Figure 8. All of the samples show the characteristic bands of Nafion at about 385 cm^−1^ [δ(CF_2_], 731 cm^−1^ [ns(CF_2_)], 804 cm^−1^ [n(C–S)], 971 cm^−1^ [ns(C–S)], 1059 cm^−1^ [ns(SO_3_^−^)], 1212 cm^−1^ [nas(CF_2_)], 1295 cm^−1^ [n(C–C)] and 1375 cm^−1^ [ns(C–C)] [30]. No significant shift of the Nafion peaks was detected in composite membranes with respect to the pure one. Likewise, the peaks of TiO_2_ anatase incorporated within the composite membranes spectra occur at the same wavenumbers as in the S-TiO_2_ powder. Moreover, a spatial inhomogeneity, over the scale of 10 μm, of the S-TiO_2_ distribution within the membranes, was present in all the nanocomposite samples, so that the spectra reported in Figure 8 should be considered as the representative spectra of the three composite membranes.

**Figure 9:**
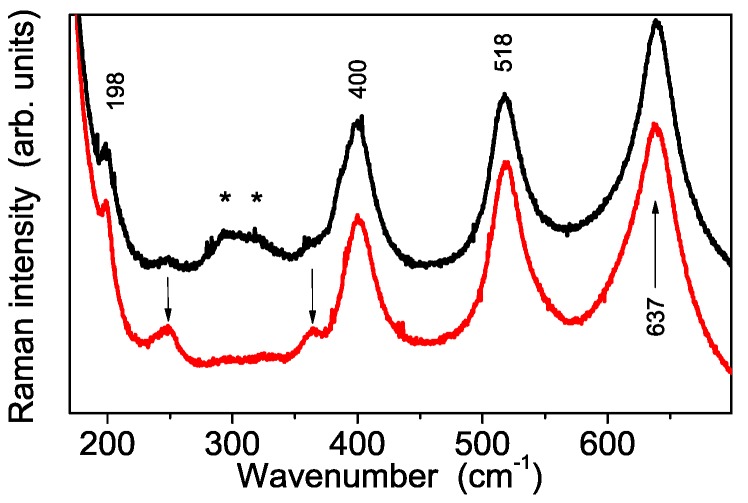
Experimental Raman spectra carried out in the low wavenumber region on S-TiO_2_ nanopowder sample (red trace) and on nanocomposite membrane loaded by 7% *w*/*w* of S-TiO_2_ (black trace). The two arrows indicate the TiO_2_-B phase modes at about 250 and 310 cm^−1^, respectively, while the pair of stars (*) labels the Nafion peaks at about 292 cm^−1^ (t(CF_2_)) and 310 cm^−1^, assigned to the t(CF_2_) mode [25].

As for the Raman spectrum of sulfate groups, shown after proper magnification in the inset of Figure 8, a weak peak occurring at about 1000 cm^−1^ is clearly observed in composite membranes heavily loaded by S-TiO_2_ (*i.e*., with 5% and 7% *w*/*w*), thus confirming the persistence of sulfate groups in composite samples after the powder incorporation. Moreover, the comparison between the Raman spectra carried out in the low wavenumber region from S-TiO_2_ nanopowders and from composite membranes, see Figure 9, indicates the occurrence of an important phase rearrangement of the TiO_2_ component incorporated into the Nafion membrane, which results in a remarkable decrease of the TiO_2_-B phase, while the anatase one seems to be unaffected.

**Figure 10:**
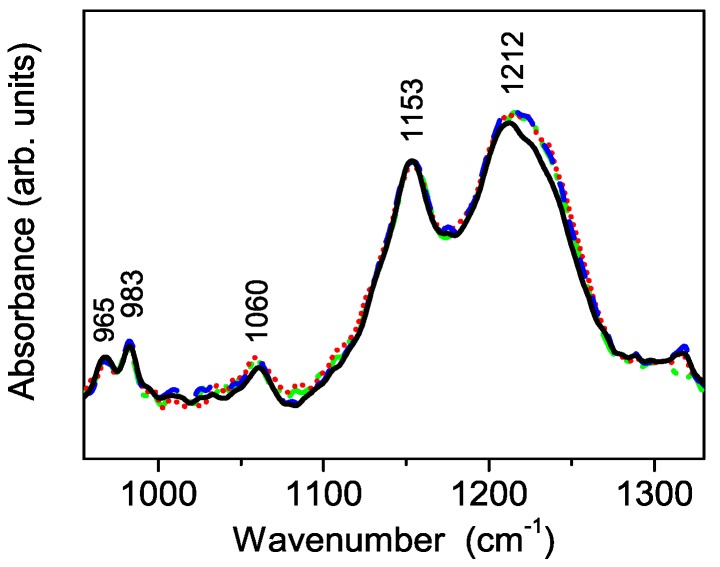
FT-IR ATR spectra of the pure Nafion (black line) and loaded composite *n*-TiO_2_-S-N (*n* = 2, 5, 7) membranes showing small, but significant, differences in the spectral region near 1220 cm^−1^. For the details refer to the text.

The FT-IR ATR spectra of pure and loaded Nafion show five main peaks (Figure 10), all related to Nafion membrane, at about 965, 983, 1060, 1153, and 1212 cm^−1^, respectively. They turn out in good agreement with the assignments reported in the literature [31]. A small, but systematic, spectral difference is observed for all the loaded samples at about 1220 cm^−1^.

In principle the origin of the observed difference in the spectral shape in this region might be related either to a rearrangement of the Nafion local structure (and thus of its vibrational properties) due to the powder incorporation or, in alternative, to a contribution of the S-TiO_2_ sulfate peak at 1224 cm^−1^. However, in the latter case, one should expect to observe an additional change in the spectrum of the composite membrane near 1136 cm^−1^, related to the sulfate peak observed in powders with intensity even higher than that of the peak at 1220 cm^−1^ (see Figure 6). Unfortunately, this is not the case, so the spectral change is likewise due to a change in the Nafion local structure within the composite membrane, thus suggesting an interaction effect between filler and Nafion.

### 3.4. Membrane Morphology Related Raman Mapping

**Figure 11:**
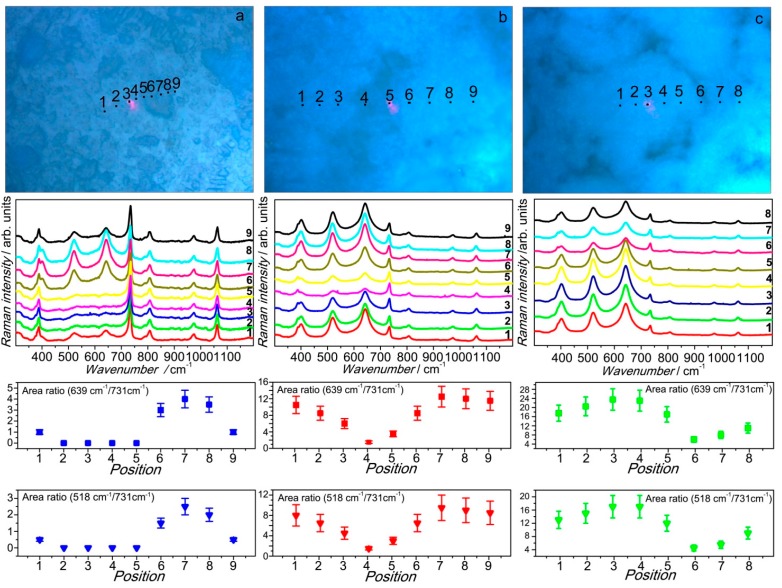
Raman mapping of the three composite membranes: 2-TiO_2_-S (**a**) 5-TiO_2_-S (**b**) and 7-TiO_2_-S (**c**). For each sample, the upper panel shows the optical microscopy image, the middle one the Raman spectra in the different samples. The red spot observed in the optical images is the laser beam, whose size is of the order of 1 micron.

Optical microscopy images of the composite membranes revealed the non-homogeneous nature of the systems on the micrometric scale. In fact, while pure Nafion looks homogenous, the filler distribution inside the Nafion membranes turns out to not be uniform independently of the incorporated amount since it originates morphologically, unlike regions inside the polymer matrix. Raman micro-spectroscopy allows for the analysis of the different micro-region in the membranes surface in order to probe the S-TiO_2_ distribution. The results of this hand-made Raman mapping, carried out from the three investigated composite membrane, are cumulatively reported in Figure 11.

Raman spectra, displayed in the three middle panels of this composite figure, were taken step-by-step, moving along a straight line on the surface of the three composite samples, respectively, from the regions serially numbered in the top panels of the figure. The obtained spectra were fitted with Lorentzian curves in order to estimate the weight of each spectral component. Afterwards, the intensity (I) ratios between the membrane peak at about 731 cm^−1^ and each of the two S-TiO_2_ peaks at about 639 and 518 cm^−1^, respectively, were determined. The choice to exploit the area of two powder peaks, in order to derive the intensity ratios, allowed to obtain two independent checks of the fitting procedure validity. The results of this analysis revealed a quite sharp correspondence between the morphology characteristics of the composite membranes and their S-TiO_2_ content, both the intensity ratios I_639_/I_731_ and I_518_/I_731_ showing the same behavior. In particular, for the two samples with higher filler content (5% and 7%) the brighter circular zones are richer in S-TiO_2_, while the surrounding areas have a lower filler amount. In contrast, in the case of the membrane loaded with 2% of filler, the filler-dense regions look darker, due to a different image contrast. Moreover, it was possible to assess that in the case of 2-TiO_2_-S a part of the membrane remained almost filler-free while, for the higher S-TiO_2_ percentages, the filler was present in the whole membrane surface, although not homogeneously distributed.

## 4. Conclusions

This paper presents and discusses the results of systematic investigations, carried out by means of a multi-techniques approach, on sulfated TiO_2_ nano-powders synthesized via a novel one-step method and three composite Nafion-based membranes, obtained by the incorporation of different amounts of these S-TiO_2_ powder. Both X-rays diffraction and Raman scattering measurements of sulfated nanopowders indicated the formation of an almost pure titania anatase phase with only a minor amount of TiO_2_-B phase, typical of nanometric samples. TEM microscopy revealed that the powders obtained through the one-step synthesis are composed by spherical nanoparticles with an average dimension of about 8 nm and a very sharp size distribution. Moreover, vibrational spectroscopy (Raman scattering and FT-IR ATR) allowed the analysis of the sulfate functionalization of the nanopowders either as synthesized or after the exposure to water vapor. The powder functionalization by sulfate groups was also confirmed by TGA analysis, and turn out to be still present even after a severe hydrothermal stability test. This is a crucial condition in order to exploit the use of the powder in presence of a high relative humidity environment, as it occurs in a working proton-conducting membrane fuel cell. The functionalization persistence was confirmed also after the S-TiO_2_ incorporation inside the Nafion membrane. Evidence of an interaction effect between the filler and the host polymeric network was revealed by FTIR spectroscopy for each one of the filler percentages explored, with a partial rearrangement of the Nafion local structure due to the powder incorporation. The inclusion of the inorganic fillers during the recast procedure induces at microscopic level the formation of zones with different S-TiO_2_ concentration inside the polymer matrix, although, at least for the case of 5% and 7% filler inclusion, sulfated titania seems to be present over the whole membrane surface. In the case of 2% S-TiO_2_ membrane, the surface structure consists of filler-rich regions, in form of islands, separated by areas of almost pure Nafion. This structural arrangement does not easily provide any proton percolation path, which in contrast requires a continuous filler presence, as it occurs for membranes loaded with higher inclusion content. Therefore, if the percolation mechanism is the predominant way for protons to conduct, a higher resistance would be expected for the 2% membrane compared to the 5% and 7% ones. Future investigations will be focused on the membranes’ electrochemical behavior, in the aim to verify this hypothesis and, possibly, highlight the influence of hydration on their conduction properties.

## Figures and Tables

**Table 1:** Plain Nafion and composite Nafion membranes investigated in this work.

Sample	Filler	Filler content (wt %)	Sample acronym
Plain Nafion	None	0	N
Composite Nafion	Superacid S-TiO_2_	2	2-TiO_2_-S
Composite Nafion	Superacid S-TiO_2_	5	5-TiO_2_-S
Composite Nafion	Superacid S-TiO_2_	7	7-TiO_2_-S
